# Urinary Leukotriene E4 as a Biomarker in NSAID-Exacerbated Respiratory Disease (N-ERD): a Systematic Review and Meta-analysis

**DOI:** 10.1007/s11882-022-01049-8

**Published:** 2022-11-14

**Authors:** Malcolm Marquette, Bhavesh V. Tailor, Philip C. Calder, Peter J. Curtis, Yoon Loke, Andrew M. Wilson

**Affiliations:** 1 Department of Respiratory Medicine, Norwich University Hospital, NorfolkNorwich, UK; 2 Norwich Medical School, University of East Anglia, Norwich, UK; 3 School of Human Development and Health, Faculty of Medicine, University of Southampton, Southampton, UK; 4 NIHR Southampton Biomedical Research Centre, University Hospital Southampton NHS Foundation Trust and University of Southampton, Southampton, UK; 5 Department of Nutrition and Preventive Medicine, Norwich Medical School, University of East Anglia, Norwich, UK

**Keywords:** Asthma, N-ERD, Non-steroidal anti-inflammatory respiratory disease, Aspirin-intolerance, Samter’s, Urinary leukotrienes E4

## Abstract

**Purpose of Review:**

Non-steroidal exacerbated respiratory disease (N-ERD) currently requires aspirin challenge testing for diagnosis. Urinary leukotriene E4 (uLTE_4_) has been extensively investigated as potential biomarker in N-ERD. We aimed to assess the usefulness of uLTE_4_ as a biomarker in the diagnosis of N-ERD.

**Recent Findings:**

N-ERD, formerly known as aspirin-intolerant asthma (AIA), is characterised by increased leukotriene production. uLTE_4_ indicates cysteinyl leukotriene production, and a potential biomarker in N-ERD. Although several studies and have examined the relationship between uLTE_4_ and N-ERD, the usefulness of uLTE_4_ as a biomarker in a clinical setting remains unclear.

**Findings:**

Our literature search identified 38 unique eligible studies, 35 were included in the meta-analysis. Meta-analysis was performed (i.e. pooled standardised mean difference (SMD) with 95% confidence intervals (95% CI)) and risk of bias assessed (implementing Cochrane Handbook for Systematic Reviews of Diagnostic Test Accuracy (Cochrane DTA)). Data from 3376 subjects was analysed (1354 N-ERD, 1420 ATA, and 602 HC). uLTE_4_ was higher in N-ERD vs ATA (*n* = 35, SMD 0.80; 95% CI 0.72–0.89). uLTE4 increased following aspirin challenge in N-ERD (*n* = 12, SMD 0.56; 95% CI 0.26–0.85) but not ATA (*n* = 8, SMD 0.12; CI − 0.08–0.33). This systematic review and meta-analysis showed that uLTE_4_ is higher in N-ERD than ATA or HC. Likewise, people with N-ERD have greater increases in uLTE_4_ following aspirin challenge. However, due to the varied uLTE_4_ measurement and result reporting practice, clinical utility of these findings is limited. Future studies should be standardised to increase clinical significance and interpretability of the results.

**Supplementary Information:**

The online version contains supplementary material available at 10.1007/s11882-022-01049-8.

## Introduction

NSAID-exacerbated respiratory disease (N-ERD) or aspirin exacerbated respiratory disease (AERD), formerly known as aspirin-intolerant asthma (AIA) and Samter’s triad, is a phenotype of asthma characterised by increased leukotriene production and leukotriene driven inflammation [1]. N-ERD is the name used henceforth as it is the term accepted in current clinical practice [2••].

N-ERD is clinically characterised by the presence of asthma, chronic rhinosinusitis with nasal polyposis, and exacerbation of respiratory symptoms on exposure to substances having cyclo-oxygenase 1 (COX-1) inhibiting activity [1, 3•]. The prevalence of N-ERD is reported to be 7% of asthmatics overall and approximately 15% in those who have severe asthma [4]. However, it occurs in 30–40% of those with asthma and nasal polyposis [5]. Accurate diagnosis of this asthma phenotype requires provocation testing, which involves nasal, oral, or inhaled challenge with aspirin [6, 7]. These procedures, whilst being clinically validated, do carry some inherent risks including significant bronchospasm and are thus not recommended for patients with severe airways disease. For these patients, diagnosis of N-ERD has typically relied on medical history alone, which increases the risk of misdiagnosing N-ERD, and the likelihood of providing inappropriate health management, by withholding the use of this class of medication in non-NERD individuals [2••]. Consequently, it is considered highly desirable to identify a robust, accessible, and safe biomarker of N-ERD.

Given that leukotriene status is heightened in N-ERD, there is significant interest in establishing their utility as candidate biomarkers for the diagnosis and disease/treatment monitoring in N-ERD. More specifically, urinary leukotriene E4 (uLTE_4_) excretion has been identified as a surrogate marker of leukotriene production in vivo and is preferred to other leukotrienes (e.g. Leukotrienes B_4,_ C_4,_ and D_4_), which have a short half-life and are difficult to measure [8, 9]. To this extent, Hagan et al. [10] reviewed the role of uLTE4 in the diagnosis of N-ERD in 2016. This is the only previous systematic review, of 10 studies, and showed uLTE_4_ as a biomarker for N-ERD. However, the inclusion criteria for that review [10] required the availability of primary level data to carry out the necessary analysis, and a proportion of full text manuscripts were not available to the authors.

Therefore, in this present study we sought to update the work carried out by Hagan et al. [10], whilst reviewing and analysing the broader literature on this subject to compare the baseline uLTE_4_ levels in patients with N-ERD, aspirin tolerant asthma (ATA), and healthy control (HC) subjects. In addition, we aimed to determine the impact of aspirin challenge testing on uLTE_4_ concentration in N-ERD and ATA individuals and the diagnostic accuracy of baseline uLTE_4_ measurements to predict aspirin intolerance in patients with asthma. In keeping with Hagan et al. [10], we analysed the different assays separately, given the variations in these techniques.

## Methods

### Literature Search

The protocol for the review was published in the PROSPERO database (CRD42021228674) and developed with reference to the Preferred Reporting Items for Systematic Reviews and Meta-analyses (PRISMA) 2020 guidelines [11]. A systematic search of MEDLINE, EMBASE, EMCARE, CINAHL and PsycINFO was undertaken by a medical librarian in conjunction with one reviewer (B.V.T.) from database inception to 31st December 2021. In contrast to the previous review, a comprehensive search strategy was implemented which captured all studies reporting baseline uLTE4 levels in N-ERD and ATA groups, irrespective of whether these studies reported primary level data to answer our primary research question. No filters were used. The strategies were peer reviewed by a second reviewer (M.M.) prior to final execution of the search. Reference lists from included studies and review articles that were identified through the database searches were hand searched to identify additional articles for possible inclusion. Both Healthcare Databases Advanced Search (HDAS) and Rayyan were used to identify duplicate records and additional duplicates were manually removed before screening for inclusion. Articles were screened by two independent reviewers (B.V.T., M.M.). Disagreements between the reviewers were resolved through discussion. The full search strategy can be found in Online Resource 1.

### Study Eligibility

The following medical diagnosis terminologies, i.e. N-ERD/AERD, Samter’s triad, and AIA, have been interchangeably used in the literature to describe the population of interest and were included within the search criteria to ensure completeness of data capture and synthesis.

Original research studies recruiting human subjects with asthma utilising uLTE_4_ as a biomarker (*index test*) to differentiate N-ERD from ATA were considered for inclusion. Diagnosis of N-ERD required at least one of the following two criteria to be met (*reference standard*): (a) positive aspirin challenge, either historic (case–control study design) or performed prospectively (singe-gate design); (b) unequivocal history of asthma exacerbation following ingestion of aspirin and/or other NSAIDs. There were no age restrictions.

The following exclusion criteria were applied: publication types other than primary studies (review articles, case reports, conference abstracts, book chapters and letters to the editor); papers published in languages other than English if a translation could not be found. Studies concerning aspirin challenge testing of asthmatic patients were excluded if baseline (pre-challenge) uLTE_4_ data was not reported in the published article, in supplementary material, or on request from the corresponding author of the publication.

### Study Outcomes

The primary study outcome was to determine whether uLTE_4_ concentration at baseline in N-ERD is different from ATA and (non-asthmatic) HC subjects, using a between-group comparison. Secondary outcomes were (a) to determine the diagnostic accuracy of baseline uLTE4 measurements to predict aspirin intolerance in patients with asthma; and (b) to determine the change in uLTE_4_ concentration in N-ERD and ATA following aspirin challenge testing.

### Data Extraction

Two reviewers (B.V.T., M.M.) independently extracted the following data from included studies: author(s); year of publication; country of origin; source of funding; demographic characteristics (*n*, sex, age); clinical characteristics (inclusion/exclusion criteria, co-morbidities, definition of asthma, baseline pulmonary function); index test (method of uLTE_4_ analysis, original units, nature of urine collection); reference standard (clinical history/aspirin challenge/both, criteria for N-ERD); mean and standard deviation (SD) of uLTE_4_ at baseline for N-ERD, ATA and HC; diagnostic test accuracy (if reported—area under curve, cut-off value, sensitivity, specificity, positive predictive value, negative predictive value); mean and SD of uLTE_4_ following aspirin challenge testing for N-ERD and ATA (if performed). Two attempts at requesting missing data from the corresponding authors of included studies were made by contacting them via e-mail. Disagreements in data extraction were resolved through discussion.

If relevant data concerning baseline and/or post-challenge uLTE_4_ were presented in published figures but not specified as summary data in the accompanying text or supplementary materials, the underlying numerical data was extracted from relevant figures using WebPlotDigitizer (v4.4, California, USA), a web-based semi-automated extraction tool [12].

### Risk of Bias Assessment

A modified version of the QUADAS tool from the Cochrane Handbook for Systematic Reviews of Diagnostic Test Accuracy was used to assess the methodological quality of included studies [13]. This was performed independently by two reviewers (B.V.T., M.M.), with disagreements resolved through discussion.

### Data Synthesis and Meta-analysis

A descriptive synthesis of included studies was performed and structured around the review objectives. Studies reporting the mean and SD of uLTE_4_ at baseline (± post-challenge) for N-ERD, ATA, and HC were included in our meta-analysis. If the extracted data were described as the median with range, or the median with interquartile range, then the data were converted to mean and SD using established approximation methods [14]. Data presented in separate subgroups were combined using established formulae from the Cochrane Handbook for Systematic Reviews of Interventions [15]. Pooled standardised mean difference (SMD) and 95% confidence intervals (CI) were calculated. We investigated the presence of statistical heterogeneity among included studies by using the *I*^2^ test. The random-effects model was used if there was significant heterogeneity (*I*^2^ > 50%), otherwise the fixed-effects model was used to combine the results. To explore possible sources of heterogeneity, meta-regression analysis was performed, with variables including publication year, country of study origin, sample size, male percentage, and baseline lung function. Any *p* values of < 0.05 were considered statistically significant.

In a change to the planned data synthesis as registered in PROSPERO, summary receiver-operating characteristic (SROC) modelling was not performed since individual data points were largely missing from included studies. Hence, evaluation of test diagnostic accuracy was not possible.

All data were extracted and stored in an Excel data file (Microsoft Excel for Mac; Microsoft Corporation, USA). Review Manager version 5.4 (The Cochrane Collaboration, Copenhagen, Denmark) and R software version 4.0.1 (R Foundation for Statistical Computing, Vienna, Austria) were used for conducting the meta-analysis.

## Results

### Study Selection

**Fig. 1:**
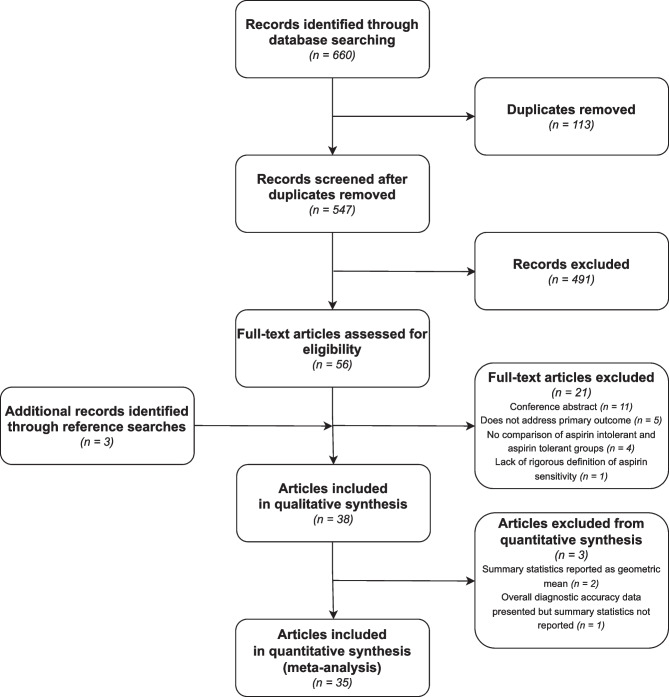
Flowchart showing process of article selection for inclusion

A total of 660 articles were identified [December 2021], with 547 article titles and abstracts reviewed following de-duplication. Of these, 491 articles were ineligible for full-text review. A total of 38 eligible full-text articles were reviewed (Fig. 1). Each article described a unique study. We performed qualitative synthesis of all included studies (*n* = 38) and meta-analysis of 35 studies. Three of the studies which did not meet the criteria for inclusion in the meta-analysis did not have the required effect size data to allow for such an analysis.

### Study Characteristics

Included studies (*n* = 38) were published between 1991 and 2021, across 8 countries [study numbers as follows: Japan (*n* = 13), Poland (*n* = 11), USA (*n* = 5), South Korea (*n* = 3), Sweden (*n* = 2), United Kingdom (*n* = 2), Italy (*n* = 1), Switzerland (*n* = 1)]. A total of *n* = 1354 N-ERD, *n* = 1420 ATA, and *n* = 602 HC subjects were represented across the included studies, with *n* = 1010 (36.5%) males. In 19 studies, patients with N-ERD were study-defined N-ERD and/or there was clear documentation concerning co-morbid chronic rhinosinusitis and/or nasal polyposis status. In the remaining studies (*n* = 19), the terminology AIA was used without reference to presence of nasal polyposis. The main characteristics of included studies are summarised in Table 1.

**Table 1:** Summary characteristics of included studies (*n* = 38)

Study	Country of origin	N-ERD study no	ATA study no	Controls	Age, N-ERD (y^a^)	Age, ATA (y^a^)	Gender, male (%)	Subjects	Definition of asthma	Lung function, FEV_1_ (%)
Ban et al. 2016 [39]	South Korea	45	44	N/A	40.3 (13.4)	45.6 (13.5)	32.6%	NS	ATS criteria	N-ERD [mean, SD] = 84.7 (17.9) ATA [mean, SD] = 86.3 (16.2)
Ban et al. 2021 [49•]	South Korea	47	90	20	51.8 (11.9)	49.4 (16.2)	35.0%	Exclusion: treatment with type 2 biologics within 130 days of enrollment; current smokers or recent ex-smokers; controller medication change within 7 days of enrollment	GINA guidelines	N-ERD [mean, SD] = 90.0 (19.5) ATA [mean, SD] = 90.7 (16.9)
Bochenek et al. 2003 [25]	Poland	65	66	50	41.6 (12.4)	34.6 (12.9)	38.9%	Stable asthma Exclusion: exacerbation or LRTI in preceding 6 weeks	NS	N-ERD [mean, SD] = 84.9 (14.3) ATA [mean, SD] = 92.5 (14.5)
Bochenek et al. 2018 [8]	Poland	247	239	95	49.3 (12.9)	49.3 (14.8)	30.9%	Stable asthma Exclusion: exacerbation in preceding 6 weeks	NS	N-ERD [mean, SD] = 80.0 (19.9) ATA [mean, SD] = 87.0 (19.8)
Cahill et al. 2015 [41]	USA	29	10	N/A	47.3 (9.9)	36.3 (3.3)	41%	Non-smoker; N-ERD group consisted of subjects undergoing aspirin desensitization	Physician-diagnosed	N-ERD [mean, SD] = 84.4 (13.4) ATA [mean, SD] = 91 (6)
Cahill et al. 2019 [42]	USA	40	13	N/A	47.0 (9.2)	34.4 (15.3)	38.1%	Stable asthma; non-smoker Exclusion: exacerbation requiring hospitalisation in preceding 6 weeks; pregnancy; breast-feeding; severe GORD, peptic ulcer, GI bleed or bleeding diathesis; antiplatelet or anticoagulant medication	Physician-diagnosed	N-ERD [mean, SD] = 91.2 (12.5) ATA [mean, SD] = 86.7 (10.9)
Choi et al. 2021 [50•]	South Korea	34	25	N/A	44.5 (10.3)	49.2 (19.1)	27.1%	NS	NS	N-ERD [mean, SD] = 86.6 (20.3) ATA [mean, SD] = 94.5 (15.3)
Christie et al. 1991 [44]	UK	6	5	N/A	31–55	24–30	36.4%	NS	NS	N-ERD [mean, SD] = 89 (16.4) ATA [mean, SD] = 93 (10.3)
Christie et al. 1992	Switzerland	6	6	N/A	44.2 (6.9)	35.5 (11.4)	25%	NS	NS	N-ERD [mean, SD] = 78.3 (9.9) ATA [mean, SD] = 85.5 (7.7)
Comhair et al. 2018 [9]	USA	240	226	71	49.3 (12.4)	49.7 (15.0)	30.5%	Stable asthma Exclusion: exacerbation in preceding 6 weeks	NS	N-ERD [mean, SD] = 79.8 (20.1) ATA [mean, SD] = 86.6 (21.0)
Gaber et al. 2008 [27]	Sweden	11	10	N/A	46 (35–63)	45.5 (27–56)	33.3%	Stable asthma; non-smoker; suspicion of NSAID intolerance	NS	≥ 70%
Higashi et al. 2002 [17]	Japan	13	10	N/A	54.8 (9.6)	52.5 (16.2)	56.5%	Stable asthma; non-smoker Exclusion: LRTI in preceding 6 weeks	ATS criteria; GINA guidelines	N-ERD [mean, SD] = 77.8 (19.3) ATA [mean, SD] = 75.3 (16.0)
Higashi et al. 2003 [18]	Japan	64	73	35	53.3 (21–79)	51.2 (21–80)	44.5%	Stable asthma Exclusion: cystic fibrosis; immotile cilia syndrome; autoimmune disease; LRTI in preceding 6 weeks	ATS criteria	N-ERD [mean, SD] = 77.3 (19.8) ATA [mean, SD] = 80.7 (21.5)
Higashi et al. 2010 [28]	Japan	10	7	N/A	45.1 (24–64)	59.4 (24–73)	11.8%	Adult subjects; suspicion of NSAID intolerance Exclusion: URTI in preceding 6 weeks; renal or liver dysfunction; hypertension; autoimmune disease	ATS criteria; GINA guidelines	N-ERD [mean, SD] = 80.2 (12.7) ATA [mean, SD] = 81.9 (14.3)
Jerschow et al. 2016 [29]	USA	16	13	N/A	37.8 (12.8)	42.6 (8.7)	41.4%	NS	Physician-diagnosed	N-ERD [mean, SD] = 73.0 (12.4) ATA [mean, SD] = 92.5 (33.9)
Kawagishi et al. 2002 [19]	Japan	48/60^b^	51/100^b^	33/110^b^	54.1 (12.4)	50 (17)	42.5%	Stable asthma Exclusion: prescribed leukotriene receptor antagonist; LRTI in preceding 6 weeks	ATS criteria	NS
Kumlin et al. 1992 [45]	Sweden	9	15	N/A	NS	NS	NS	NS	NS	NS
Laidlaw et al. 2012 [43]	USA	10	9	8	45 (20–65)	37 (22–76)	39.3%	Non-smoker	Physician-diagnosed	N-ERD [mean, SD] = 82 (9) ATA [mean, SD] = 88 (15)
Mastalerz et al. 2001 [30]	Poland	11	32	16	47.5 (10.1)	37.5 (14.3)	44.2%	Stable asthma	NS	≥ 70%
Mastalerz et al. 2002a [31]	Poland	26	33	N/A	44.6 (29–61)	45.8 (20–67)	28.8%	NS	NS	N-ERD [mean, SD] = 72.3 (12.7) ATA [mean, SD] = 69.3 (14.3)
Mastalerz et al. 2002b [48]	Poland	19	21	N/A	40.8 (23–60)	35.4 (19–60)	62.5%	Stable asthma	NS	N-ERD [mean, range] = 85.3 (64.4–113.6) ATA [mean, range] = 86.3 (61.0–111.6)
Mastalerz et al. 2008 [32]	Poland	19	21	30	42.4 (13.3)	43.6 (12.5)	40%	Stable asthma Exclusion: exacerbation or LRTI in preceding 6 weeks	NS	≥ 70%
Mastalerz et al. 2015 [33]	Poland	28	25	N/A	46.1 (14.0)	43.8 (11.5)	47.2%	Stable asthma Exclusion: exacerbation or LRTI in preceding 6 weeks	GINA guidelines	N-ERD [median, IQR] = 99.1 (15.6) ATA [median, IQR] = 98 (17.1)
Micheletto et al. 2006 [34]	Italy	67	51	N/A	41.8 (11.9)		40.7%	Mild to moderate asthma; non-smoker; suspicion of aspirin intolerance and/or NP and/or CRS Exclusion: total obstruction of ≥ 1 nostril (inability to perform NPT)	NS	Mean (SD) = 80.1 (5.8)
Mita et al. 2001 [20]	Japan	10	10	N/A	50.3 (16.4)	46.8 (17.2)	25%	Stable asthma	NS	≥ 70% (except for 1 patient in ATA group)
Mita et al. 2004 [35]	Japan	7	6	18	49.9 (19.4)	45.5 (18.0)	53.8%	Stable asthma	NS	N-ERD [mean, SD] = 82.5 (14.3) ATA [mean, SD] = 99.2 (21.4)
Mitsui et al. 2015 [21]	Japan	30	21	14	52 (13)	53 (17)	19.6%	Stable asthma	ATS criteria	N-ERD [mean, SD] = 89 (20) ATA [mean, SD] = 92 (19)
Obase et al. 2001 [46]	Japan	7	7	N/A	39.7 (12.1)	35.9 (10.3)	35.7%	Stable asthma; non-smoker Exclusion: LRTI in preceding 6 weeks	NHLBI criteria	N-ERD [mean, SD] = 89.8 (5.8) ATA [mean, SD] = 90.7 (7.8)
Obase et al. 2002 [47]	Japan	6	7	N/A	29.5 (6.2)	39.9 (11.9)	30.8%	Stable asthma; non-smoker Exclusion: LRTI in preceding 6 weeks	NHLBI criteria	≥ 80%
Ono et al. 2011 [36]	Japan	15	11	10	51 (42–65)	55 (38–68)	38.5%	Stable asthma; non-smoker	ATS criteria; GINA guidelines	N-ERD [median, range] = 71.6 (65.5–96.0) ATA [median, range] = 88.5 (61.2–98.2)
Oosaki et al. 1997 [22]	Japan	22	17	10	NS	NS	48.7%	Exclusion: history of smoking; severe asthma attack on study day; renal or liver dysfunction; ischaemic heart disease; autoimmune disease	ATS criteria	NS
Pezato et al. 2016 [37]	Poland	20	18	N/A	46 (19)	44 (19)	26.3%	NS	GINA guidelines	N-ERD [mean, SD] = 94.2 (15.8) ATA [mean, SD] = 88.3 (9.2)
Sanak et al. 2004 [38]	Poland	14	20	10	41.4 (13.9)	36.5 (12.3)	64.7%	Stable asthma Exclusion: exacerbation in preceding 6 weeks	NS	N-ERD [mean, SD] = 81.5 (12.5) ATA [mean, SD] = 92.6 (14.9)
Sanak et al. 2010 [16]	Poland	41	83	50	44.5 (21–66)		37.1%	NS	NS	NS
Smith et al. 1992 [56]	UK	10	31	17	21–54	18–34	75.6%	NS	Clinical history; reversibility	N-ERD [mean, SD] = 97 (10) ATA [mean, SD] = 86 (15)
Swierczynska-Krepa et al. 2014 [40]	Poland	20	14	N/A	46 (19)	49.5 (15)	29.4%	Aged 18–65 Exclusion: history of life-threatening anaphylactic reactions precipitated by NSAIDs; autoimmune disease; severe systemic disease; neoplasm; pregnancy	GINA guidelines	N-ERD [median, IQR] = 88.7 (17.8) ATA [median, IQR] = 92.5 (30.9)
Yamaguchi et al. 2011 [23]	Japan	15	16	10	53.9 (16.0)	59.2 (20.3)	45.2%	Adult subjects Exclusion: LRTI in preceding 6 weeks; cardiovascular disease; renal or liver dysfunction	ATS criteria	N-ERD [mean, SD] = 81.7 (16.9) ATA [mean, SD] = 88.0 (20.1)
Yamaguchi et al. 2016 [24]	Japan	15	15	28	51.1 (14.5)	50.6 (13.3)	33.3%	Stable asthma; CRS Exclusion: URTI in preceding 6 weeks; cystic fibrosis; immotile cilia syndrome; Churg-Strauss syndrome; autoimmune disease	ATS criteria	NS

*ATA* aspirin-tolerant asthma, *ATS* American Thoracic Society, *CRS* chronic rhinosinusitis, *FEV*_*1*_ forced expiratory volume in one second, *GI* gastrointestinal, *GINA* Global Initiative for Asthma, *GORD* gastro-oesophageal reflux disease, *IQR* interquartile range, *LRTI* lower respiratory tract infection, *N/A* not applicable, *N-ERD* NSAIDs exacerbated respiratory disease, *NHLBI* National Heart, Lung, and Blood Institute, *NP* nasal polyposis, *NPT* nasal provocation test, *NS* not specified, *NSAID* non-steroidal anti-inflammatory drug, *SD* standard deviation, *URTI* upper respiratory tract infection

^a^Ages may be reported as median (IQR), median (range), mean (SD), mean (range), or range

^b^Ratio represents the number of participants with basal uLTE_4_ data reported compared to the overall number of participants recruited

Across all the studies included in this review, uLTE_4_ concentration was measured using one of 4 different techniques: (i) Amersham-enzyme immunoassay (A-EIA) (*n* = 8), (ii) Cayman-enzyme immunoassay (C-EIA) (*n* = 18), (iii) mass spectrometry (MS) (*n* = 7), and (iv) radioimmunoassay (RIA) (*n* = 6), with Sanak et al. reporting results with both C-EIA and MS (thus represented twice in these overview data) [16].

Twenty-seven studies used positive aspirin challenge alone (inhaled, intravenous, nasal, or oral) as the reference standard to diagnose N-ERD, two studies used convincing clinical history of asthma exacerbation secondary to ingestion of aspirin alone, and the remaining nine studies used either positive challenge or convincing clinical history. Further details on the aspirin challenge criteria and methodology for uLTE_4_ measurement are found in Table 2.

**Table 2:** Challenge criteria and methodology of uLTE_4_ analysis in included studies (*n* = 36)

Study	Reference standard	Challenge agent	Challenge undertaken?	Criteria for N-ERD	Method of uLTE_4_ analysis	Original units of uLTE_4_	Urine sampling
Ban et al. 2016 [39]	Challenge or positive history	Lysine aspirin inhalation	Retrospectively	Fall in FEV_1_ of ≥ 20% relative to baseline	MS	pmol/mg Cr	Spot urine
Ban et al. 2021 [49]	Challenge or positive history	Lysine aspirin inhalation	Retrospectively	Fall in FEV_1_ of ≥ 20% relative to baseline	MS	pg/mg Cr	Spot urine
Bochenek et al. 2003 [25]	Challenge	Oral aspirin	Retrospectively	Fall in FEV_1_ of ≥ 20% relative to baseline	C-EIA	pg/mg Cr	Spot urine
Bochenek et al. 2018 [8]	Challenge or positive history	NS	Retrospectively	Asthma exacerbation precipitated by NSAID administration	C-EIA	pg/mg Cr	Spot urine
Cahill et al. 2015 [41]	Positive history	N/A	N/A	Characteristic reactions upon ingestion of COX-1 inhibitors	MS	pmol/mg Cr	Spot urine
Cahill et al. 2019 [42]	Challenge	Oral aspirin	Retrospectively	NS	MS	ng/mg Cr	Spot urine
Choi et al. 2021 [50•]	Challenge	Lysine aspirin inhalation	Retrospectively	NS	MS	ng/mg Cr	Spot urine
Christie et al. 1991 [44]	Challenge	NS	Retrospectively	Fall in FEV_1_ of ≥ 15% relative to baseline	RIA	pg/mg Cr	Spot urine × 2 (10 days apart)
Christie et al. 1991 [44]	Challenge	Oral aspirin	Retrospectively	Fall in FEV_1_ of ≥ 15% relative to baseline	RIA	pg/mg Cr	Spot urine × 2 (1 week apart)
Comhair et al. 2018 [9]	Challenge or positive history	NS	Retrospectively	Asthma exacerbation precipitated by NSAID administration	C-EIA	pg/mg Cr	Spot urine
Gaber et al. 2008 [27]	Challenge	Lysine aspirin inhalation	Prospectively	Fall in FEV_1_ of ≥ 20% compared with post-saline FEV_1_	C-EIA	ng/mmol Cr	Spot urine
Higashi et al. 2002 [17]	Challenge or positive history	NS	Retrospectively	Asthma exacerbation precipitated by NSAID administration	A-EIA	pg/mg Cr	Spot urine
Higashi et al. 2003 [18]	Challenge or positive history	NS	Retrospectively	Severe bronchoconstriction and nasal symptoms precipitated by ingestion of ≥ 2 different NSAIDs	A-EIA	pg/mg Cr	Spot urine
Higashi et al. 2010 [28]	Challenge	Lysine aspirin	Prospectively	Fall in FEV_1_ of ≥ 20% relative to baseline	C-EIA	pg/mg Cr	Spot urine
Jerschow et al. 2016 [29]	Challenge	Oral aspirin	Prospectively	Fall in FEV_1_ of ≥ 20% relative to baseline	C-EIA	pg/mg Cr	Spot urine
Kawagishi et al. 2002 [19]	Challenge or positive history	NS	Retrospectively	Asthma exacerbation precipitated by NSAID administration	A-EIA	pg/mg Cr	Spot urine
Kumlin et al. 1992 [45]	Challenge or positive history	NS	Retrospectively	NS	RIA	ng/mmol Cr	Spot urine
Laidlaw et al. 2012 [43]	Challenge	Oral aspirin	Retrospectively	Fall in FEV_1_ of ≥ 15% relative to baseline	MS	ng/mg Cr	Spot urine
Mastalerz et al. 2001 [30]	Challenge	Lysine aspirin inhalation	Retrospectively	NS	C-EIA	pg/mg Cr	Spot urine
Mastalerz et al. 2002a [31]	Challenge	Lysine aspirin inhalation; oral aspirin	Retrospectively	NS	C-EIA	pg/mg Cr	Spot urine
Mastalerz et al. 2002b [48]	Challenge	Oral aspirin	Retrospectively	NS	C-EIA	pg/mg Cr	Spot urine
Mastalerz et al. 2008 [32]	Challenge	Oral aspirin	Retrospectively	NS	C-EIA	pg/mg Cr	Spot urine
Mastalerz et al. 2015 [33]	Challenge	Oral aspirin	Retrospectively	NS	C-EIA	pg/mg Cr	Spot urine
Micheletto et al. 2006 [34]	Challenge	Lysine aspirin nasal	Prospectively	Nasal resistance increased > 40% in at least one nostril relative to baseline; volume of one nostril decreased > 10% from baseline	C-EIA	pg/mg Cr	Spot urine
Mita et al. 2001 [20]	Challenge	Lysine aspirin intravenous	Prospectively	Fall in FEV_1_ of ≥ 20% relative to baseline	A-EIA	pg/mg Cr	Spot urine
Mita et al. 2004 [35]	Challenge	Lysine aspirin intravenous	Prospectively	Fall in FEV_1_ of ≥ 20% relative to baseline	C-EIA	pg/mg Cr	Spot urine
Mitsui et al. 2015 [21]	Challenge	Lysine aspirin inhalation; oral aspirin	Retrospectively	NS	A-EIA	pg/mg Cr	Spot urine
Obase et al. 2001 [46]	Challenge	Oral aspirin	Prospectively	Fall in FEV_1_ of ≥ 20% relative to baseline	RIA	pg/mg Cr	Spot urine
Obase et al. 2002 [47]	Challenge	Oral aspirin	Prospectively	Fall in FEV_1_ of ≥ 20% relative to baseline	RIA	pg/mg Cr	Spot urine
Ono et al. 2011 [36]	Challenge	NS	Retrospectively	NS	C-EIA	pg/ml Cr	Spot urine
Oosaki et al. 1997 [22]	Positive history	N/A	N/A	History of aspirin sensitivity	A-EIA	pg/mg Cr	Spot urine
Pezato et al. 2016 [37]	Challenge	Oral aspirin	Prospectively	Fall in FEV_1_ of ≥ 20% relative to baseline	C-EIA	pg/ml Cr	Spot urine
Sanak et al. 2004 [38]	Challenge	Oral aspirin	Retrospectively	Fall in FEV_1_ of ≥ 20% relative to baseline	C-EIA	pg/mg Cr	Spot urine
Sanak et al. 2010 [16]	Challenge	NS	Retrospectively	NS	C-EIA; MS	pg/mg Cr	Spot urine
Smith et al. 1992 [56]	Challenge	NS	Retrospectively	Fall in FEV_1_ of ≥ 15% relative to baseline	RIA	pg/mg Cr	Spot urine
Swierczynska-Krepa et al. 2014 [40]	Challenge	Oral aspirin	Prospectively	Fall in FEV_1_ of ≥ 20% relative to baseline	C-EIA	pg/mg Cr	Spot urine
Yamaguchi et al. 2011 [23]	Challenge	Lysine aspirin intravenous	Retrospectively	Fall in FEV_1_ of ≥ 20% relative to baseline	A-EIA	pg/mg Cr	Spot urine
Yamaguchi et al. 2016 [24]	Challenge or positive history	NS	Retrospectively	Asthma exacerbation precipitated by NSAID administration	A-EIA	pg/mg Cr	Spot urine

*A-EIA* Amersham-enzyme immunoassay, *C-EIA* Cayman-enzyme immunoassay, *COX-1* cyclooxygenase-1, *FEV*_*1*_ forced expiratory volume in one second, *MS* mass spectrometry, *N-ERD* NSAIDs exacerbated respiratory disease, *NSAID* non-steroidal anti-inflammatory drug, *RIA* radioimmunoassay, *uLTE4* urinary leukotriene E4, *N/A* not applicable, *NS* not specified

### Key Findings

**Fig. 2:**
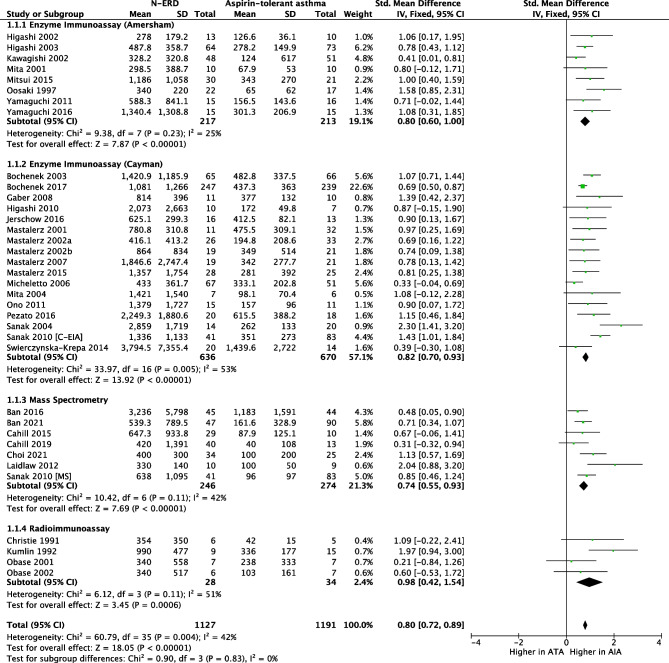
Forest plot of baseline uLTE_4_ for N-ERD vs ATA [35 studies]

**Fig. 3:**
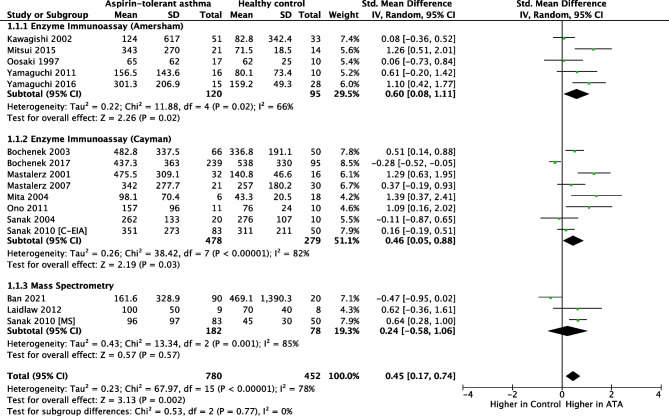
Forest plot of baseline uLTE_4_ for ATA vs HC [15 studies]

**Fig. 4:**
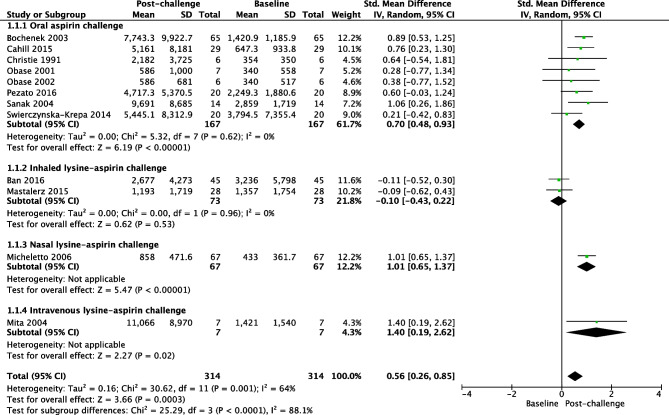
Forest plot of uLTE_4_ pre- and post-aspirin challenge in N-ERD [12 studies]

**Fig. 5:**
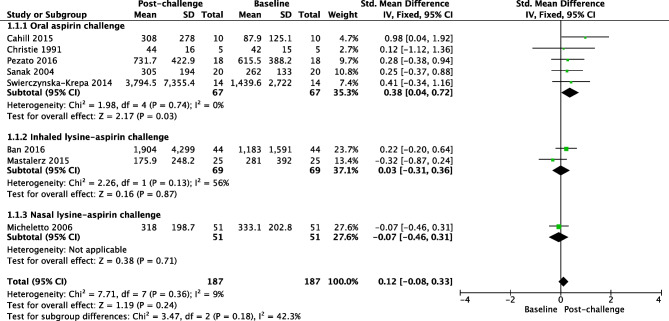
Forest plot of uLTE_4_ pre- and post-aspirin challenge in ATA [8 studies]

Studies with different uLTE_4_ measurement methodologies were combined. Thirty-five studies including 1127 N-ERD and 1191 ATA reported that the baseline concentration of uLTE_4_ was significantly higher in N-ERD (SMD 0.80, 95% CI = 0.72 to 0.89; *I*^2^ = 42%, Fig. 2) [16–46, 47, 48, 49•, 50•]. Fifteen studies including 780 ATA and 452 HC reported that the baseline concentration of uLTE_4_ was significantly higher in ATA (SMD 0.45, 95% CI = 0.17 to 0.74; *I*^2^ = 78%, Fig. 3) [16, 19, 21–26, 30, 32, 35, 36, 38, 43, 49•]. The concentration of uLTE_4_ increased following aspirin challenge in N-ERD (12 studies, *n* = 314 SMD 0.56; 95% CI = 0.26 to 0.85, Fig. 4) [25, 33–35, 37–41, 44, 46, 47] but not ATA (8 studies, *n* = 187, SMD 0.12; 95% CI =  −0.08 to 0.33, Fig. 5) [16, 19, 21–26, 30, 32, 35, 36, 38, 43].

### Meta-regression and Risk of Bias

Heterogeneity observed between studies in this meta-analysis was low. Despite this, we performed meta-regression analysis to assess the contribution of several covariates on effect size across studies included in pooling of effect size for baseline uLTE_4_ in N-ERD vs ATA comparison. *I*^2^ for this analysis was low (42%). Meta-regression revealed that country of study had an impact on effect size (*I*^2^ = 13.05%). Furthermore, by identifying different study sites and including this in the multiple regression analysis, we found that this would account for an *I*^2^ of 100%, suggesting that heterogeneity across studies in this meta-analysis is related to site. There was no significant impact on the effect size when other covariates (publication year, percentage male participants, baseline lung function, and methodology for uLTE_4_ measurement) were analysed by means of meta-regression, and hence no significant impact on heterogeneity between studies was noted.

**Fig. 6:**
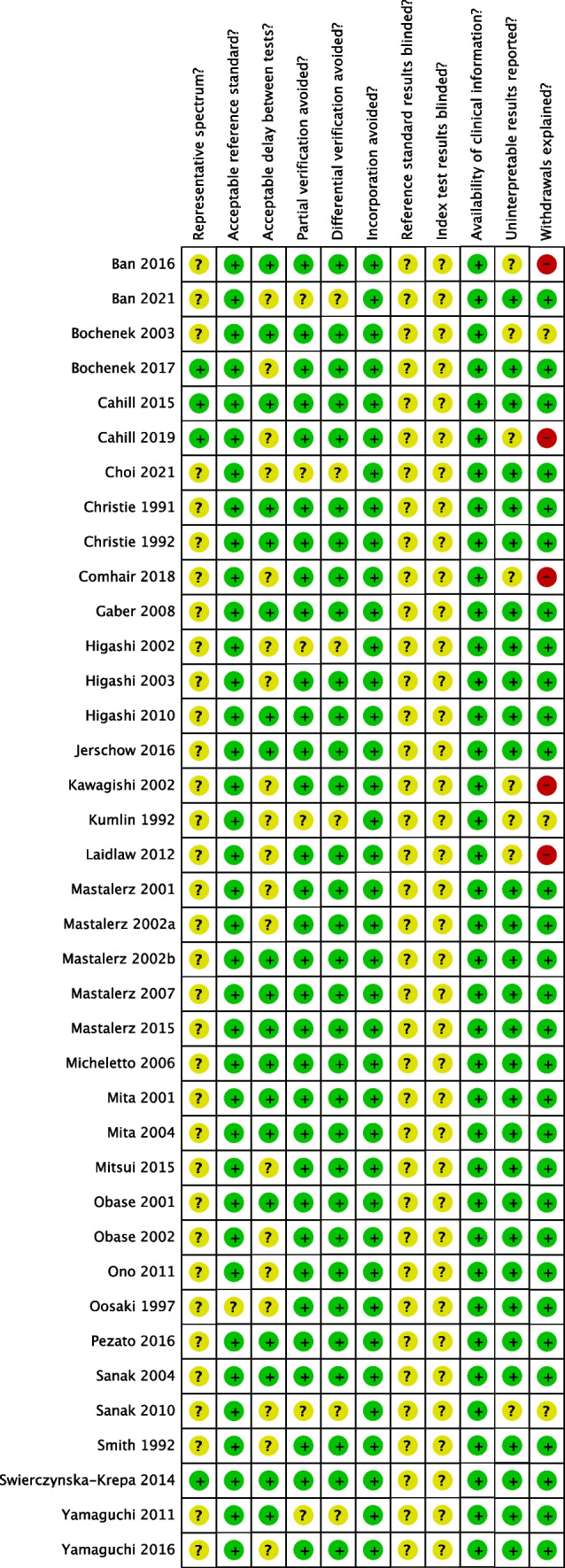
Risk of bias summary

**Fig. 7:**
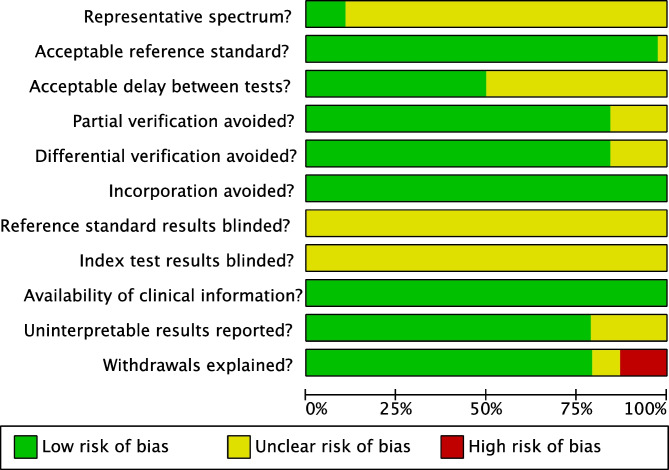
Risk of bias graph

Risk of bias assessed by means of the QUADAS tool from the Cochrane Handbook for Systematic Reviews of Diagnostic Test Accuracy [13], was acceptable across all studies; however 37.8% of quality assessment items were unfulfilled (Figs. 6 and 7). The following risk of bias items were poorly reported across all studies (reported in < 30% overall): spectrum of representative patients (10.5%) and independent interpretation of index and reference standard tests (0%).

## Discussion

Our meta-analysis of 35 studies demonstrated a statistically significant higher baseline concentration of uLTE_4_ in patients with N-ERD compared to those with ATA and HC, adding an addition 25 studies to the previous review. These findings corroborate current knowledge regarding the importance of leukotriene status in patients with N-ERD, and again identify uLTE_4_ as a potential biomarker in N-ERD diagnosis and disease monitoring. For the subset of studies reporting uLTE_4_ measurements before and after aspirin challenge testing, a significant rise in uLTE_4_ was seen in patients with N-ERD, but not those with ATA. This is the first meta-analysis which evaluates the change in uLTE_4_ concentrations following aspirin challenge in N-ERD compared to ATA, and the results are consistent with previous literature demonstrating that the magnitude of nasal and/or respiratory reactions to provocative aspirin challenges in asthmatics is associated with both the degree of baseline uLTE_4_ elevation and the rise in uLTE_4_ during a challenge [51, 52].

This study has a number of limitations. Because individual data points were largely missing from most studies, sensitivity and specificity testing was not possible. Four studies did provide some data of interest [8, 9, 16, 38], but this was insufficient to carry out this analysis. The corresponding authors of the rest of the included studies were contacted via e-mail asking for this data, but there was no response from any of them. Studies included were published between 1991 to 2021, a total span of 30 years, and this will invariably carry with it a variation in practice of uLTE_4_ measurement. Although, our meta-regression analysis did not identify year of publication as contributing to heterogeneity across studies, four different methodologies were used to measure uLTE_4_ across the studies included. However, to account for this, a separate comparison analysis for studies using each of the methods was performed and then the studies were combined. This analysis has revealed that despite the different methodologies, there was no significant heterogeneity across studies (Fig. 2), meaning that different methodologies were not shown to have a significant impact on effect size. Although the different methodologies did not appear to result in heterogeneity, there was a large number of methodologies used and methods of reporting the data. The country of publication had an effect on heterogeneity but not when site was included in the multiple regression. This suggests that site was responsible for the heterogeneity, presumably due to a composite of methodology, definition of N-ERD and population sampled. Greater standardisation of the procedure and reporting is required in clinical research and clinical practice.

There was also variation in the way asthma was defined across studies, with American Thoracic Society (ATS) criteria, Global Initiative for Asthma (GINA) guidelines, National Heart, Lung and Blood Institute criteria, and physician diagnosis all used. In 17 studies, definition of asthma was not specified. This is important given that it will dictate the characteristics of the population being studied. Similarly, the definition of aspirin intolerance varied across studies. Although most studies performed aspirin challenge testing (either retrospectively or prospectively), there was considerable variation in the challenge agent employed and the diagnostic cut-off for a positive test (i.e., fall in FEV_1_ relative to baseline). Approximately half of studies included in the meta-analysis (18/35) provided clear documentation of co-morbid chronic rhinosinusitis and/or nasal polyposis status, or the aspirin-intolerant cohort was defined as N-ERD. The remaining studies did not provide such population characteristics. In several studies, summary data concerning uLTE_4_ levels were not stated in the published text or supplementary materials and had to be derived from figures using a web-based extraction tool. This invariably is an estimation of the data. Similarly, for studies where the reported data was described as median with range or interquartile range, this required conversion to mean and SD using published approximation methods. This is important because of the potential impact this has on the accuracy of the results and the impact this could have on the weight of the individual studies, and therefore the overall study results. We therefore feel that standardisation of result reporting should also be implemented.

One of the most important features of this meta-analysis is the enforced use of the standardised mean difference. This summary statistic is used when the measurement scales of the various papers are too diverse to be pooled in a meta-analysis, and thus they have to be converted to a common statistical denominator, or statistical units. The use of the standardised difference means that we cannot know the absolute difference between groups, nor can we define a diagnostic cut off. This is important especially when considering developing study protocols going forward with the aim of establishing sensitivity and specificity. This work has identified the need for standardisation of such protocols to move closer towards achieving clinical significance. Our results show that all the methodologies employed to measure uLTE_4_ yielded comparable results across studies. Mass spectrometry has been described in a number of publications as the gold standard for the measurement of leukotrienes in biological fluids [53, 54]; however, access to MS and cost might impact its availability in the clinical setting, whereas, enzyme immunoassays might be more readily available. We feel that these are important considerations to make going forward in the protocol development for research of this subject area. This would allow calculation of the absolute mean difference in clinically useful terms rather than the slightly abstract concept of a standardised mean difference. The current heterogeneity in methods and measurement makes it impossible to come up with clinically relevant recommendations on the use of such diagnostic technology.

It should also be noted that most studies have been conducted in specialist centres and excluded participants with uncontrolled asthma or participants reporting a respiratory tract infection or asthma exacerbation in the preceding 6 weeks. While this provides a well-defined cohort for research purposes, our findings may not be generalisable to patients undergoing testing in routine clinical practice, especially since N-ERD is most prevalent among patients with severe asthma.

Overall, the risk of bias was acceptable across all studies. However, in all included studies, it was not reported whether study authors were blinded to baseline uLTE_4_ data (*index test*) when performing aspirin challenge testing or obtaining clinical history of aspirin intolerance (*reference standard*). The primary aim of many included studies was not to determine test diagnostic accuracy, which may account for this. It is also unclear how much a lack of blinding could affect interpretation of aspirin challenge testing since challenges are normally undertaken following a set protocol with a pre-determined diagnostic cut-off.

The finding of a significant rise in uLTE4 following aspirin challenge testing is in keeping with the central role leukotriene release as a cause of upper and lower airway symptoms [44]. Daffern et al. showed that rise in uLTE4 following challenge was related to severity of airflow obstruction post challenge. However interestingly the rise does not seem to be attenuated by inhibition of 5-lipoxygenase which should reduce leukotriene production [51, 55].

## Conclusion

The true prevalence of N-ERD is unclear and it is likely to be significantly underdiagnosed especially in those individuals with mild respiratory symptoms, and because of difficulty accessing specialist centres for diagnostic confirmation [2••, 4]. An accurate diagnosis of N-ERD is important, as this can have an impact on both treatment modalities and management of co-morbid chronic diseases such as ischaemic heart disease and chronic pain. Including uLTE_4_ in the diagnostic algorithm for patients suspected to suffer from N-ERD would be especially useful in individuals who may be at higher risk of adverse reactions from aspirin challenge testing because of increased risk such as FEV_1_ < 70%, or nasal pathology (precluding nasal aspirin challenge test) [2••]. This safe, non-invasive biomarker for N-ERD may reduce clinician time needed for aspirin challenge testing and would be cost-effective. Future research should be directed at evaluating diagnostic specificity and sensitivity to establish biomarker diagnostic accuracy and employing standardised methods of uLTE_4_ measurements to ensure any results yielded are more readily translatable to impact clinical practice.

## Supplementary Information

Below is the link to the electronic supplementary material.

Supplementary file1 (PDF 112 KB)
