# Supplementary material for: Urinary Leukotriene E4 as a Biomarker in NSAID-Exacerbated Respiratory Disease (N-ERD): a Systematic Review and Meta-analysis
Source: Curr Allergy Asthma Rep. 2022 Nov 14;22(12):209–29. doi: 10.1007/s11882-022-01049-8 (PMC9732072; doi:10.1007/s11882-022-01049-8)
Supplement: Supplementary file 1 — Supplementary file1 (PDF 112 KB) [file 11882_2022_1049_MOESM1_ESM.pdf]

Article title: Urinary leukotriene E4 as a biomarker in NSAID-exacerbated respiratory disease (N-ERD): a systematic review and meta-analysis

Journal name: Current Allergy and Asthma Reports

Author names: Malcolm Marquette, Bhavesh V Tailor, Philip C Calder, Peter J Curtis, Andrew M Wilson

Corresponding author: Dr Malcolm Marquette, Department of Respiratory Medicine, Norfolk & Norwich University Hospital, Norwich, UK; M.Marquette@uea.ac.uk

Online Resource 1 Detailing the search strategy

Searches performed using Healthcare Databases Advanced Search (HDAS) – 05/01/2021

The MEDLINE search strategy is listed below:

#	Database searched	Search term	Results
1	MEDLINE	"LEUKOTRIENE E4"/	959
2	MEDLINE	"LEUKOTRIENE E4"/ur	309
3	MEDLINE	(ulte4).ti,ab	32
4	MEDLINE	(lte4).ti,ab	1109
5	MEDLINE	(Leukotriene ADJ (E4 OR "E 4")).af	1191
6	MEDLINE	(1 OR 3 OR 4 OR 5)	1741
7	MEDLINE	(ur).fs OR (urine).af OR (urinary).af	673670
8	MEDLINE	(6 AND 7)	579
9	MEDLINE	(2 OR 8)	579
10	MEDLINE	exp ASTHMA/ OR NASAL POLYPS/ OR (sampters triad).af	135337
11	MEDLINE	asa OR aspirin* OR aerd OR (acetylsalicyl*).af	101422
12	MEDLINE	(9 AND 11)	122
13	MEDLINE	asthma* OR (polyp*).af	326391
14	MEDLINE	(9 AND 13)	330
15	MEDLINE	(9 AND 10)	279
16	MEDLINE	(12 OR 14 OR 15)	344
17	MEDLINE	(12 OR 14 OR 15) [Languages English]	318
